# Electrochemical Detection of Morphine in Untreated Human Capillary Whole Blood

**DOI:** 10.1021/acsomega.1c00773

**Published:** 2021-04-21

**Authors:** Elsi Verrinder, Niklas Wester, Elli Leppänen, Tuomas Lilius, Eija Kalso, Bjo̷rn Mikladal, Ilkka Varjos, Jari Koskinen, Tomi Laurila

**Affiliations:** † Department of Electrical Engineering and Automation, Aalto University, Tietotie 3, Espoo 02150, Finland; ‡ Department of Chemistry and Materials Science, Aalto University, Kemistintie 1, Espoo 02150, Finland; § Department of Pharmacology, University of Helsinki, Haartmaninkatu 8, Helsinki 00290, Finland; ∥ Department of Clinical Pharmacology, University of Helsinki and Helsinki University Hospital, Tukholmankatu 8C, Helsinki 00290, Finland; ⊥ Emergency Medicine, University of Helsinki and Department of Emergency Medicine and Services, Helsinki University Hospital, Helsinki 00014, Finland; # Department of Anesthesiology, Intensive Care and Pain Medicine, University of Helsinki and Helsinki University Hospital, Haartmaninkatu 2A, Helsinki 00290, Finland; ∇ Canatu Oy, Tiilenlyöjänkuja 9, Vantaa 01720, Finland

## Abstract

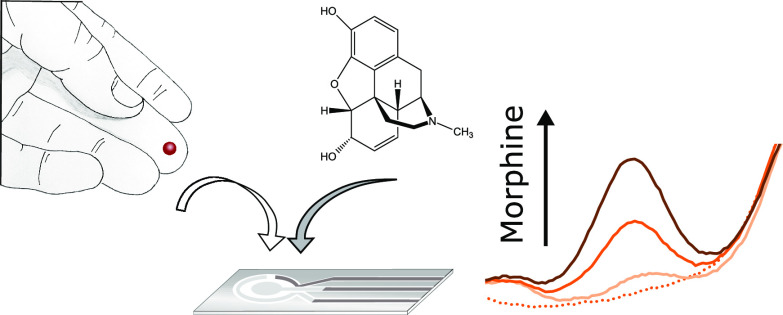

Disposable single-use electrochemical sensor strips were used for quantitative detection of small concentrations of morphine in untreated capillary whole blood. Single-walled carbon nanotube (SWCNT) networks were fabricated on a polymer substrate to produce flexible, reproducible sensor strips with integrated reference and counter electrodes, compatible with industrial-scale processes. A thin Nafion coating was used on top of the sensors to enable direct electrochemical detection in whole blood. These sensors were shown to detect clinically relevant concentrations of morphine both in buffer and in whole blood samples. Small 38 μL finger-prick blood samples were spiked with 2 μL of morphine solution of several concentrations and measured without precipitation of proteins or any other further pretreatment. A linear range of 0.5–10 μM was achieved in both matrices and a detection limit of 0.48 μM in buffer. In addition, to demonstrate the applicability of the sensor in a point-of-care device, single-determination measurements were done with capillary samples from three subjects. An average recovery of 60% was found, suggesting that the sensor only measures the free, unbound fraction of the drug. An interference study with other opioids and possible interferents showed the selectivity of the sensor. This study clearly indicates that these Nafion/SWCNT sensor strips show great promise as a point-of-care rapid test for morphine in blood.

## 1 Introduction

Morphine is a strong opioid used for treatment of moderate to severe pain, especially in chronic cancer pain.^1^ Unfortunately, morphine like all opioids, can also cause addiction, overdose, and even death due to respiratory depression. According to a report from the Centers for Disease Control and Prevention, over 46,000 opioid-related overdose deaths were recorded in the United States in 2018.^2^ The ability to monitor the patients’ blood concentrations of morphine would enable personalized, safe dosing of the opioid and fast diagnosis in cases of overdose. However, there are currently no such tools available for detection of morphine.

The standard method for determining opioid concentrations from biological samples is high-performance liquid chromatography (HPLC) often coupled with mass spectrometry.^3−5^ While this method offers extremely low detection limits and high accuracy, the analysis has to be performed in a fully equipped laboratory, requires professional personnel to run, and takes up to several hours to complete. In contrast, electrochemical methods can be used to develop point-of-care (POC)-type devices that provide quantitative measurements of drug concentrations in blood at patient’s bedside. These methods have fast response times of a few minutes, sufficiently low detection limits, and simple instrumentation.

Electrochemical sensors have been developed for the detection of hundreds of analytes, including opioids and other drugs, in both buffer solution and biological matrices, such as urine,^6−10^ blood plasma,^11−13^ serum,^14,15^ and even whole blood.^16^ However, in the vast majority of these studies the common approach for conducting the measurements is treating the samples with several protocols to remove proteins and dilute the sample for a facilitated detection. In addition, most studies only show detection of unrealistically high concentrations of opioids in recovery studies.

Urine is the most commonly used biological matrix for recovery studies. However, in many clinical situations, urine is not the preferable matrix to be used for POC detection due to practical reasons. On the other hand, if plasma or serum is used as the measurement matrix, they have to be extracted from the original whole blood samples, resulting in even more complex and time-consuming pretreatment protocols that are not acceptable in emergency situations such as suspected overdose cases. Using a simple finger-prick blood sample without the need for any additional treatment steps would be ideal for a rapid POC test for opioids.

**Figure 1:**
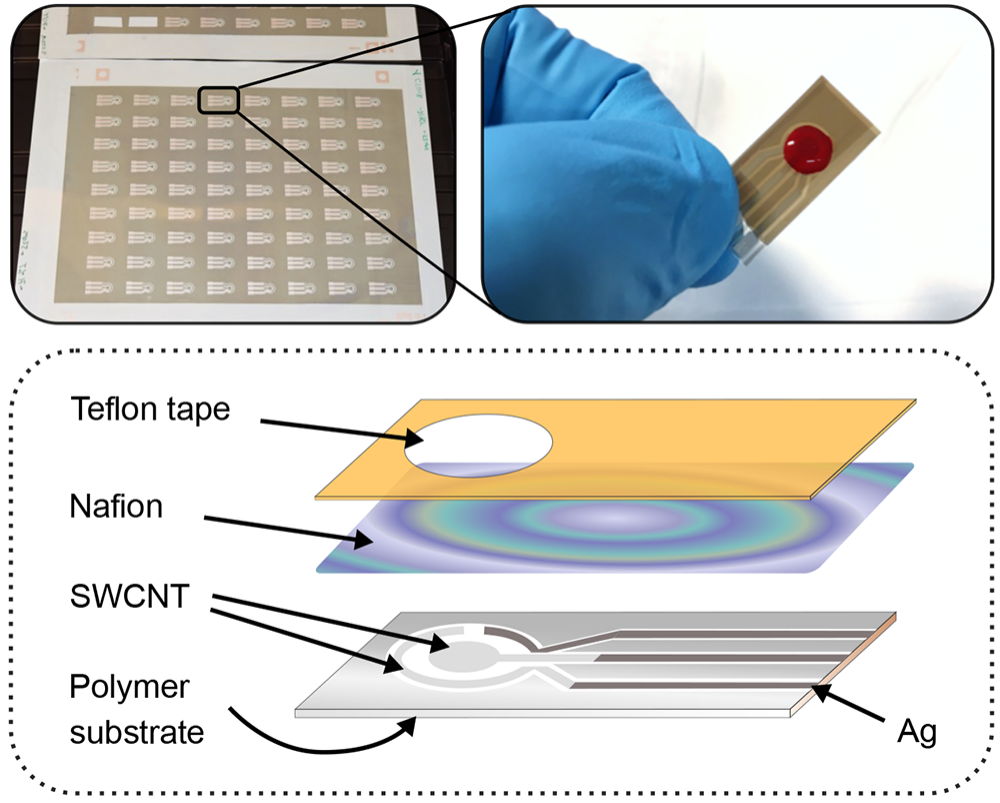
Photos of the sensor sheet on the polymer substrate, a close-up of the prepared sensor, and a schematic figure of the layered sensor structure.

In this work, we use single-walled carbon nanotube (SWCNT) network sensor strips for the detection of morphine in untreated capillary whole blood (Figure 1). SWCNT networks are an attractive material for use in electrochemical sensors due to their high surface area, conductivity, and compatibility with industrial manufacturing processes. We have recently shown that these SWCNT networks can be fabricated into disposable electrochemical sensor strips that can be used for single determinations of analytes in biological matrices.^17^

A permselective Nafion coating is used on top of the electrodes. Nafion is a copolymer consisting of a hydrophobic backbone and negatively charged sulfonic side groups that form hydrophilic channels with a few nanometers in diameter.^18^ This structure gives Nafion its cation-exchange properties allowing cations to permeate through the film while blocking anionic molecules from the electrode surface.^19^ In addition, cationic molecules, especially hydrophobic ones, have been seen to enrich on Nafion-coated electrodes, thus further improving the sensitivity of the sensor.^20^ In our previous studies, we have seen that Nafion can be used to improve the selectivity and sensitivity of the electrode toward analgesics, including morphine, in biological matrices.^17,21,22^

This paper presents detection of clinically relevant concentrations of morphine in the nM range in buffer and in untreated, undiluted capillary whole blood with a small sample volume of 40 μL. Furthermore, to demonstrate the applicability of the Nafion/SWCNT sensor as a POC device, we show successful single-point determinations of small concentrations of morphine in finger-prick blood samples from three volunteers.

## 2 Results and Discussion

### 2.1 Characterization of Nafion/SWCNT Strips

The structure of SWCNT networks synthesized in a similar method have been previously characterized in refs (22,23,24). Extensive characterization was done with ultraviolet–visible spectroscopy (UV–vis), X-ray photoelectron spectroscopy (XPS), energy-dispersive spectroscopy (EDS), and X-ray absorption spectroscopy (XAS). The main results from these experiments are summarized in Table 1. It should be noted that most of these results have been obtained from SWCNT networks with an optical transparency of about 90% (at 550 nm). Briefly, the mean diameter of the SWCNTs was determined with UV–vis and was seen to have an average of 2.1 nm.

**Table 1:** Summary of the Characterization of the SWCNT Networks^a^

sheet resistance (two sheets)^b^	optical transparency (at 550 nm, two sheets)^c^	UV–vis^d^	XPS^d^	EDS^d^	XAS^d^
53.6 ± 1.2 Ω/sq 69.6 ± 3.6 Ω/sq	55.2 ± 0.4% 66.5 ± 0.4%	mean SWCNT diameter 2.1 nm	C 71.7 ± 0.2 at% O 8.7 ± 0.2 at% Si^e^ 19.5 ± 0.3 at% Fe 0.1 ± 0.01 at%	catalyst particles: C and Fe SWCNT sidewall: C	highly sp^2^-bound carbon with a clear long-range order ketone/aldehyde and carboxyl peaks detected
					iron particles: iron carbide and iron oxide

a Results obtained in this and previous work.^23,24^

b Values given as an average of five measurement points with standard deviations as errors.

c Error given as a typical standard deviation between 36 sheets and 341 measurement points.

d Measured from SWCNT networks with an optical transparency of ∼90% (550 nm) and a sheet resistance of 88 Ω/sq.

e Most of the detected oxygen and all silicon from the native oxide of the silicon wafer substrate.

XPS showed that the nanotubes consisted of 71.7 ± 0.2 at% carbon, 8.7 ± 0.2 at% oxygen, and 0.1 ± 0.01 at% iron. Si (19.5 ± 0.3 at%) was also detected, but most of this and the detected oxygen are most likely from the partially showing Si wafer and native oxide on the wafer. The XAS studies revealed a high sp^2^ fraction and long-range order and the presence of surface functional groups. The values for sheet resistance for the two sheets fabricated in this study were reported to be 53.6 ± 1.2 Ω/sq and 69.6 ± 3.6 Ω/sq and the optical transparencies at 550 nm were 66.5 ± 0.4 and 55.2 ± 0.4%, respectively.

Additional characterization of similarly fabricated Nafion/SWCNT strips was conducted in a study by Wester et al.^17^ The thicknesses of the SWCNT/Nafion layer (fabricated with 2.5% Nafion) and the Ag reference were studied with SEM from cross-sectional samples. These samples revealed the composite nature of the SWCNT/Nafion layer, showing a mixture of the two materials on the polymer substrate. The total thickness of this composite layer including a very thin (∼65–75 nm) layer of only Nafion on top was found to be approximately 170 nm. The thickness of the silver reference was 5.9–7.2 μm.

Electrochemical characterization of the integrated SWCNT test strips showed close to reversible electron transfer.^17^ This was studied with an outer-sphere redox probe hexaammineruthenium(III) chloride (Ru(NH_3_)_6_^2+/3+^), with which a Δ*E*_p_ of 68.8 mV was obtained. The effective measurement range of these sensors was up to 1.1 V (vs Ag). It was also shown that the Nafion layer significantly stabilized the Ag quasi-reference electrode in PBS preventing the potential from drifting over time. Moreover, it should be noted that while the Nafion layer used on these sensor strips is thick enough to protect the reference electrode, it is still thin enough not to significantly influence the time resolution of the sensor.^25,26^ This is an essential property for sensors designed for rapid POC devices. The main electrochemical properties of the SWCNT sensor strips are listed in Table 2.

**Table 2:** Electrochemical Characterization of SWCNT Networks^a^,^b^

property	electrode	method	result
Δ*E*_p_ in 1 mM Ru(NH_3_)_6_^2+/3+^ (100 mV/s)	Nafion/SWCNT on PET	CV	68.8 mV
effective measurement range in PBS	Nafion/SWCNT on PET	DPV	up to 1.1 V (vs Ag)
Ag reference stability in PBS	Ag reference on PET	E vs Ag/AgCl	drift of >60 mV
(for 2 h)			
	Nafion-coated Ag reference on PET	E vs Ag/AgCl	no drift

a The SWCNT networks had an optical transparency of 71.6% (at 550 nm) and a sheet resistance of 73 Ω/sq.

b Results obtained from our previous work with an integrated SWCNT sensor strip coated with 2.5% Nafion.^17^

### 2.2 Calibration in Phosphate Buffered Saline (PBS) and Capillary Blood

**Figure 2:**
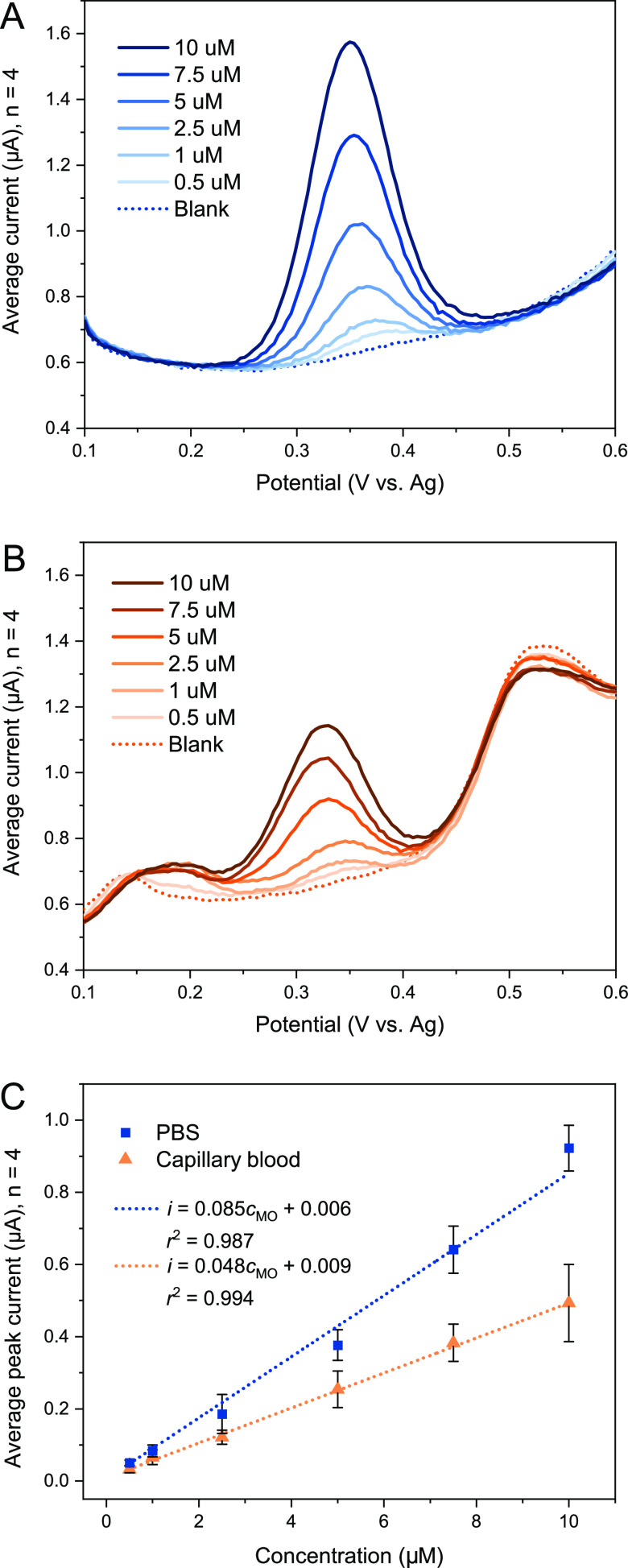
Average DPV curves of linear calibrations of morphine. Increasing concentrations of morphine measured in (A) PBS (pH 7.4) and (B) untreated capillary blood (minimal dilution due to spiking). The linear calibration curves are shown in (C) with standard deviations as error bars (*n* = 4). All measurements are done with the Nafion/SWCNT strip with 5 min accumulation time.

The differential pulse voltammetry (DPV) curves for increasing concentrations of morphine are shown in Figure 2 both in PBS (A) and whole blood (B). Both graphs show the average DPV curves for four electrodes. The current peak seen for morphine at around 0.35 V is commonly attributed to the oxidation of the phenol group at the 3-carbon.^27^Figure 2C shows the calibration curves with background-subtracted peak currents with standard deviations as error bars. A linear range of 0.5–10 μM was obtained for morphine both in PBS (*i =* 0.085*c*_MO_ + 0.006, *r*^2^ = 0.987) and in whole blood (*i =* 0.048*c*_MO_ + 0.009, *r*^2^ = 0.994) with a detection limit of 0.48 μM in PBS.

The detection limit obtained in this study corresponds to clinical plasma concentrations of morphine reported in cancer patients with chronic pain^28,29^ and cases of overdose.^30^Table 3 compares the current study to other reports in the literature conducted on detection of morphine in biological samples within the last 5 years.

**Table 3:** Comparative Table of the State-of-the-Art Literature from the Past 5 Years on Studies of Electrochemical Detection of Morphine in Biological Matrices^a^

electrode	LOD in PBS (μM)	linear range (μM)	biological matrix	sample treatment	lowest detected in the matrix (μM)	ref
M-CNF/CPE	0.0019	0.0033–55 55–245	serum	precipitation ((NH_4_)_2_SO_4_), centrifugation, and dilution	50	(14)
MWCNT/MgFe_2_O_4_/CPE	0.01	0.05–920	serum	precipitation (acetonitrile), centrifugation, and drying + dilution	10	(34)
CMNP–CPE	0.003	0.01–2	serum	precipitation (methanol), centrifugation, and dilution	10	(35)
		2–720				
Zn_2_SnO_4_–GO/CPE	0.011	0.02–15	plasma	precipitation (HClO_4_), centrifugation, and dilution 5×	2	(11)
Pt/PSi–CILE	0.03	0.1–25	serum	precipitation (HClO_4_), centrifugation, and dilution 5×	1	(8)
RhN-MC-modified GCE	0.04	0.1–20	serum	precipitation (methanol) and dilution	0.2	(31)
Nafion/SWCNT	0.48	0.5–10	capillary whole blood	**minimal dilution** (due to spiking)	**0.5**	this work

a GO, graphene oxide; CPE, carbon paste electrode; RhN, rhodium nanoparticle; MC, mesoporous carbon; GCE, glassy carbon electrode; MWCNT, multiwalled carbon nanotube; M-CNF, magnetic carbon nanofiber; CMNP, chitosan-coated magnetic nanoparticle; PSi, porous silicon; and CILE, carbon ionic liquid electrode

As can be seen from the table, lower theoretical detection limits in buffer have been achieved with several different electrode materials. It should be noted, however, that these detection limits do not translate to low concentrations detected in the real samples. In fact, the lowest reported concentration of morphine detected in biological samples is 0.2 μM,^31^ while others report detected levels above 1 μM.

It is also highlighted in Table 3 that in all the listed studies, the biological samples have been heavily pretreated before the measurements. Since all these studies use either plasma or serum, these have to be first obtained from whole blood samples by centrifugation, a process that takes around 10–15 min to complete.^32^ An additional minimum time of 30–60 min is required for obtaining high-quality serum samples.^33^ After this, proteins are precipitated with different agents such as ammonium hyposulfate ((NH_4_)_2_SO_4_), acetonitrile, methanol, or perchloric acid (HClO_4_) and separated by centrifugation. This is followed by dilution of the sample at least 5×, with the final dilution not always reported. These pretreatment steps lead to assay times from 45 min to more than an hour.

In contrast, in this study, morphine was measured in completely untreated capillary whole blood with only a minimal dilution due to spiking of the analyte into the sample, an assay protocol closely resembling a POC-type test. Morphine (0.5 μM) was detected in untreated capillary whole blood with a total assay time of only several minutes, a result that, to our knowledge, has not been published before.

### 2.3 Interference Study

**Figure 3:**
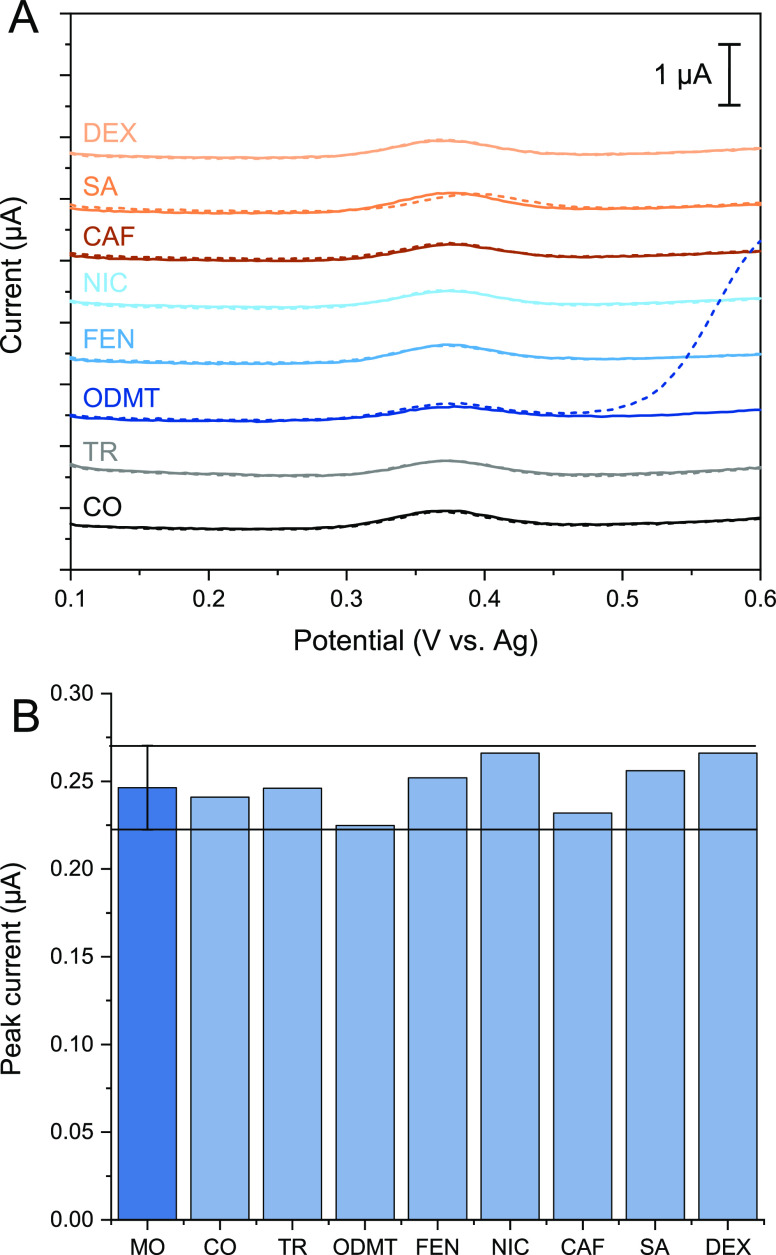
Interference study. Morphine (MO) (2.5 μM) measured in the presence of several different interferents. (A) DPV curves for (B) average peak current for 2.5 μM MO measured with eight separate electrodes (dark blue) with the RSD as tolerance limits and the corresponding peak currents for 2.5 μM MO in the presence of 10 μM codeine (CO), 10 μM tramadol (TR), 10 μM *O*-desmethyltramadol (ODMT), 2.5 μM fentanyl (FEN), 10 μM nicotine (NIC), 20 μM caffeine (CAF), 950 μM salicylic acid (SA), and 10 μM dextromethorphan (DEX). Peak currents are background-subtracted.

DPV curves for the interference measurements in PBS are shown in Figure 3A. The solid lines show the DPV curves for 2.5 μM morphine in PBS and the dashed lines 2.5 μM morphine in the presence of the interferent. Thus, it is shown that the addition of the interferent does not affect the oxidation peak of morphine.

In Figure 3B, the average peak current for 2.5 μM morphine measured with eight separate sensors is shown as the dark blue bar and a relative standard deviation (RSD) of 9.9% determines the toleration limits shown as horizontal lines. This figure shows that none of the studied interferents cause a change in the morphine peak current greater than the tolerance limits. Thus, the molecules tested here do not interfere with the detection of morphine.

### 2.4 Recovery Study in Capillary Blood

Since this test strip is designed for single-use determination of morphine in untreated capillary whole blood, a recovery test was carried out with capillary blood from three different subjects. The samples were spiked with three concentrations of morphine (1, 2.5, and 5 μM) and measured with separate sensors as single determinations. Samples were only minimally diluted due to spiking. The measurements were conducted without fasting, on different days, and different times of the day.

The measured single-point determinations are listed in Table 4. The detected morphine concentrations were in good agreement with the linear calibrations and the average RSD of the three concentrations between the three subjects was 7.7%. An average recovery of morphine in capillary whole blood was found to be 60% with slight variations between subjects. The unbound fraction of morphine in blood at room temperature is known to be about 53–69%,^36−40^ which suggests that the Nafion/SWCNT sensor only detects the free fraction of morphine.

**Table 4:** Recovery Studies from Three Different Subjects

	added (μM)	found (μM)	recovery %	RSD % (*n* = 3)
subject 1	1	0.56	56.2	
	2.5	1.65	65.8	
	5	2.57	51.4	
subject 2	1	0.55	55.4	
	2.5	1.39	55.5	
	5	3.03	60.5	
subject 3	1	0.67	66.7	
	2.5	1.69	67.4	
	5	3.03	60.7	
				
average			60.0	7.7

We have seen the same behavior before in our previous work with Nafion-coated SWCNT networks on glass electrodes.^22^ In this previous study, a recovery of 61.4% was found for morphine and 41.5% for codeine in plasma. The unbound fraction of codeine has been shown to be 44–45%.^38,39^ Similar results were also obtained in another study by Wester et al.^17^ where the same sensor structure was applied in the detection of acetaminophen in capillary whole blood.

The same study verified that these sensor strips are resistant to passivation and thus the decrease in the current in whole blood versus PBS cannot be explained by simple passivation by proteins.^17^ It is likely that this resistance to protein fouling is due to the antifouling properties of Nafion, also observed in other studies.^41^

In a recent study on the detailed structure of Nafion thin films, the diameter of the nanoscale channels extending throughout the film were found to be between 3–6 nm.^42^ On the other hand, the dimensions of the most abundant protein in human blood, albumin, are about 3.8 nm in diameter and 15 nm in length.^43^ Thus, it is conceivable that most proteins—as well as any opioid molecules bound to them—are excluded from the Nafion-coated electrode surface, protecting the surface from biofouling.

The results obtained in this work demonstrate the vital role of hybrid materials in sensor design. While the SWCNT network provides the necessary sensitivity toward the target analyte, the Nafion coating enriches cationic analytes thus further improving sensitivity and is essential for providing both selectivity and protection against fouling for successful measurements in biological matrices.

## 3 Conclusions

In this work, we demonstrated quantitative electrochemical detection of clinically relevant concentrations of morphine in untreated capillary whole blood. We used a disposable SWCNT network sensor strip fabricated on a flexible polyethylene terephthalate (PET) substrate and coated with a thin Nafion membrane. With these sensors, a linear range of 0.5–10 μM was achieved in both PBS and whole blood and a detection limit of 0.48 μM was obtained in PBS. In addition, single-determination measurements were done with finger-prick blood samples from three volunteers spiked with low levels of morphine. An average recovery of 60% and RSD of 7.7% suggests that the sensor only detects the free fraction of the analyte.

The complete assay time for these determinations was only several minutes and the required sample volume 40 μL. The samples were completely untreated except for a minimal dilution resulting from spiking of the analyte. The interferent study demonstrated that the detection of morphine is not hindered by other opioids and common interferents potentially present in blood. As a conclusion, these results show that the Nafion/SWCNT sensor strip has real potential to be applied in a POC device for detection of morphine in whole blood samples.

## 4 Materials and Methods

### 4.1 Production of Nafion/SWCNT Strips

The SWCNT networks were first collected on a filter by aerosol chemical vapor deposition. The deposition process is described in more detail in refs (44) and (45). The synthesized networks were then press-transferred onto an A4-sized PET substrate, densified with isopropanol, and baked at 100 °C for 10 min. To form reference electrodes and contact pads, silver was screen-printed on top of the collected SWCNT network.

The SWCNT counter and working electrodes were then laser-patterned from the SWCNT sheet. Lastly, the patterned strips were coated with 5% Nafion 117 (Sigma) with a slot-die coater (FOM Technologies). The fabricated strips were then cut out from the sheet by hand and wrapped in poly(tetrafluoroethylene) (PTFE, Saint-Gobain Performance Plastics CHR 2255–2) tape with a 6 mm diameter hole to define the measurement area. A step-by-step fabrication process is provided in ref (17). Two sheets were fabricated with a total of 144 sensor strips.

### 4.2 Electrochemical Measurements

All electrochemical measurements were performed with a PalmSens4 portable potentiostat with a 40 μL sample size. DPV was used for all measurements with a measurement window of 0.1–0.6 V, pulse amplitude 70 mV, step potential 4 mV, and pulse period 0.02 s. Before measurements with any analyte, six DPV backgrounds were first run in PBS (1×, pH 7.4) without accumulation time followed by four backgrounds with a 5 min accumulation time either in PBS or in whole blood. All measurements with an analyte were recorded with a 5 min accumulation time.

### 4.3 Calibration in PBS and Capillary Blood

Calibration measurements in PBS (0.01 M, pH 7.4) and untreated capillary whole blood were carried out with physiologically meaningful morphine concentrations of 0.5, 1, 2.5, 5, 7.5, and 10 μM with four separate electrodes in both matrices. Concentrated morphine solution (2 μL) (prepared from morphine hydrochloride, University Pharmacy, Helsinki, Finland) was spiked in 38 μL of samples, either PBS or whole blood, resulting in a minimal dilution of the sample. The electrode strip was rinsed with di-ionized water between each concentration measurement. The limit of detection (LOD) in PBS was calculated with the formula LOD = 3.3 × σ/*s*, where σ is the standard deviation of three consecutive background currents in μA and *s* is the sensitivity of the electrode (μA/μM). The value was reported as the average of four electrodes.

### 4.4 Interference Study

To assess the performance of the Nafion/SWCNT strip in the presence of potential interferents, 2.5 μM morphine was measured with eight different interferents: 10 μM codeine (codeine hydrochloride, University Pharmacy, Helsinki, Finland), 10 μM tramadol (Tramal 50 mg/mL, Orion Pharma, Finland), 10 μM *O*-desmethyltramadol (*O*-desmethyltramadol hydrochloride, Sigma), 2.5 μM fentanyl (fentanyl citrate, 50 μg/mL, Hameln), 10 μM nicotine (Sigma), 20 μM caffeine (Sigma), 950 μM salicylic acid (Sigma), and 10 μM dextromethorphan (dextromethorphan hydrobromide monohydrate, Sigma). These molecules were selected either based on their similar molecular structure to morphine or the high probability of their simultaneous presence in the blood with morphine. Each interferent was measured with a separate electrode. The measurements were done following a protocol of first measuring 2.5 μM morphine in PBS, rinsing the electrode with di-water, measuring twice in PBS and finally in a mixture of 2.5 μM morphine and the interferent. All measurements with an analyte were done with a 5 min accumulation time. The RSD for 2.5 μM morphine in PBS was calculated from these single-point measurements with eight separate electrodes.

### 4.5 Recovery Study in Capillary Blood

To simulate a real POC-type measurement, single point determinations were carried out in untreated finger-prick whole blood from three healthy volunteers. Blood samples were spiked with 1, 2.5, and 5 μM morphine and measured with three individual electrodes with 5 min accumulation time. The found morphine concentration and recoveries from these measurements were calculated from the calibration curve obtained in PBS. The RSD was given as the average over all three concentrations.

## References

[ref1] Wiffen P. J.; Wee B.; Derry S.; Bell R. F.; Moore R. A. Opioids for cancer pain - an overview of Cochrane reviews (Review). Cochrane Database Syst. Rev. 2017, 7, CD012592 10.1002/14651858.CD012592.pub2. 28683172 PMC6483487

[ref2] Wilson N.; Kariisa M.; Seth P.; Smith H. IV; Davis N. L. Drug and Opioid-Involved Overdose Deaths — United States, 2017 – 2018. MMWR Morb. Mortal. Wkly. Rep. 2020, 69, 290–297. 10.15585/mmwr.mm6911a4. 32191688 PMC7739981

[ref3] Jordan P. H.; Hart J. P. Voltammetric Behaviour of Morphine at a Glassy Carbon Electrode and Its Determination in Human Serum by Liquid Chromatography With Electrochemical Detection Under Basic Conditions. Analyst 1991, 116, 991–996. 10.1039/an9911600991. 1801606

[ref4] Kirchheiner J.; Schmidt H.; Tzvetkov M.; Keulen J. T.; Lötsch J.; Roots I.; Brockmöller J. Pharmacokinetics of codeine and its metabolite morphine in ultra-rapid metabolizers due to CYP2D6 duplication. Pharmacogenomics J. 2007, 7, 257–265. 10.1038/sj.tpj.6500406. 16819548

[ref5] Grabenauer M.; Moore K. N.; Bynum N. D.; White R. M.; Mitchell J. M.; Hayes E. D.; Flegel R. Development of a quantitative LC-MS-MS assay for codeine, morphine, 6-acetylmorphine, hydrocodone, hydromorphone, oxycodone and oxymorphone in neat oral fluid. J. Anal. Toxicol. 2018, 42, 392–399. 10.1093/jat/bky021. 29554298

[ref6] Mohammadi S.; Taher M. A.; Beitollahi H. A hierarchical 3D camellia-like molybdenum tungsten disulfide architectures for the determination of morphine and tramadol. Microchim. Acta 2020, 187, 312 10.1007/s00604-020-4134-x. 32367346

[ref7] Taei M.; Hasanpour F.; Hajhashemi V.; Movahedi M.; Baghlani H. Simultaneous detection of morphine and codeine in urine samples of heroin addicts using multi-walled carbon nanotubes modified SnO2-Zn2SnO4 nanocomposites paste electrode. Appl. Surf. Sci. 2016, 363, 490–498. 10.1016/j.apsusc.2015.12.074.

[ref8] Ensafi A. A.; Abarghoui M. M.; Rezaei B. Simultaneous determination of morphine and codeine using Pt nanoparticles supported on porous silicon flour modified ionic liquid carbon paste electrode. Sens. Actuators, B 2015, 219, 1–9. 10.1016/j.snb.2015.05.010.

[ref9] Cheraghi S.; Taher M. A.; Karimi-Maleh H. A Novel Strategy for Determination of Paracetamol in the Presence of Morphine Using a Carbon Paste Electrode Modified with CdO Nanoparticles and Ionic Liquids. Electroanalysis 2016, 28, 366–371. 10.1002/elan.201500357.

[ref10] Rajaei M.; Foroughi M. M.; Jahani S.; Zandi M. S.; Nadiki H. H. Sensitive detection of morphine in the presence of dopamine with La 3+ doped fern-like CuO nanoleaves/MWCNTs modified carbon paste electrode. J. Mol. Liq. 2019, 284, 462–472. 10.1016/j.molliq.2019.03.135.

[ref11] Bagheri H.; Khoshsafar H.; Afkhami A.; Amidi S. Sensitive and simple simultaneous determination of morphine and codeine using a Zn2SnO4 nanoparticle/graphene composite modified electrochemical sensor. New J. Chem. 2016, 40, 7102–7112. 10.1039/C6NJ00505E.

[ref12] Afkhami A.; Khoshsafara H.; Bagherib H.; Madrakianaa T. Facile simultaneous electrochemical determination of codeine and acetaminophen in pharmaceutical samples and biological fluids by graphene–CoFe_2_O_4_ nanocomposite modified carbon paste electrode. Sens. Actuators, B 2014, 203, 909–918. 10.1016/j.snb.2014.07.031.

[ref13] Hassannezhad M.; Hosseini M.; Ganjali M. R.; Arvand M. A graphitic carbon nitride (g-C3N4/Fe3O4) nanocomposite: An efficient electrode material for the electrochemical determination of tramadol in human biological fluids. Anal. Methods 2019, 11, 2064–2071. 10.1039/c9ay00146h.

[ref14] Bahrami G.; Ehzari H.; Mirzabeigy S.; Mohammadi B.; Arkan E. Fabrication of a sensitive electrochemical sensor based on electrospun magnetic nanofibers for morphine analysis in biological samples. Mater. Sci. Eng. C. 2020, 106, 110183 10.1016/j.msec.2019.110183. 31753387

[ref15] Najafi M.; Sohouli E.; Mousavi F. An Electrochemical Sensor for Fentanyl Detection Based on Multi-Walled Carbon Nanotubes as Electrocatalyst and the Electrooxidation Mechanism. J. Anal. Chem. 2020, 75, 1209–1217. 10.1134/S1061934820090130.

[ref16] Moonla C.; Goud K. Y.; Teymourian H.; Tangkuaram T.; Ingrande J.; Suresh P.; Wang J. An integrated microcatheter-based dual-analyte sensor system for simultaneous, real-time measurement of propofol and fentanyl. Talanta 2020, 218, 121205 10.1016/j.talanta.2020.121205. 32797931

[ref17] Wester N.; Mikladal B. F.; Varjos I.; Peltonen A.; Kalso E.; Lilius T.; Laurila T.; Koskinen J. Disposable Nafion-Coated Single-Walled Carbon Nanotube Test Strip for Electrochemical Quantitative Determination of Acetaminophen in a Finger-Prick Whole Blood Sample. Anal. Chem. 2020, 92, 13017–13024. 10.1021/acs.analchem.0c01857. 32842738 PMC7547857

[ref18] Mauritz K. A.; Moore R. B. State of understanding of Nafion. Chem. Rev. 2004, 104, 4535–4586. 10.1021/cr0207123. 15669162

[ref19] Szentirmay M. N.; Martin C. R. Ion-exchange selectivity of Nafion films on electrode surfaces. Anal. Chem. 1984, 56, 1898–1902. 10.1021/ac00275a031.

[ref20] Chou J.; Ilgen T. J.; Gordon S.; Ranasinghe A. D.; McFarland E. W.; Metiu H.; Buratto S. K. Investigation of the enhanced signals from cations and dopamine in electrochemical sensors coated with Nafion. J. Electroanal. Chem. 2009, 632, 97–101. 10.1016/j.jelechem.2009.04.002.

[ref21] Mynttinen E.; Wester N.; Lilius T.; Kalso E.; Koskinen J.; Laurila T. Simultaneous electrochemical detection of tramadol and O-desmethyltramadol with Nafion-coated tetrahedral amorphous carbon electrode. Electrochim. Acta 2019, 295, 347–353. 10.1016/j.electacta.2018.10.148.

[ref22] Wester N.; Mynttinen E.; Etula J.; Lilius T.; Kalso E.; Kauppinen E. I.; Laurila T.; Koskinen J. Simultaneous Detection of Morphine and Codeine in the Presence of Ascorbic Acid and Uric Acid and in Human Plasma at Nafion Single-Walled Carbon Nanotube Thin-Film Electrode. ACS Omega 2019, 4, 17726–17734. 10.1021/acsomega.9b02147. 31681878 PMC6822113

[ref23] Wester N.; Mynttinen E.; Etula J.; Lilius T.; Kalso E.; Mikladal B. F.; Zhang Q.; Jiang H.; Sainio S.; Nordlund D.; Kauppinen E. I.; Laurila T.; Koskinen J. Single-Walled Carbon Nanotube Network Electrodes for the Detection of Fentanyl Citrate. ACS Appl. Nano Mater. 2020, 3, 1203–1212. 10.1021/acsanm.9b01951.

[ref24] Mynttinen E.; Wester N.; Lilius T.; Kalso E.; Mikladal B. F.; Varjos I.; Sainio S.; Jiang H.; Kauppinen E. I.; Koskinen J.; Laurila T. Electrochemical Detection of Oxycodone and Its Main Metabolites with Nafion-Coated Single-Walled Carbon Nanotube Electrodes. Anal. Chem. 2020, 92, 8218–8227. 10.1021/acs.analchem.0c00450. 32412733 PMC7735650

[ref25] Kristensen E. W.; Kuhr W. G.; Wightman R. M. Temporal characterization of perfluorinated ion exchange coated microvoltammetric electrodes for in vivo use. Anal. Chem. 1987, 59, 1752–1757. 10.1021/Ac00141a003. 3631500

[ref26] Engstrom R. C.; Wightman R. M.; Kristensen E. W. Diffusional Distortion in the Monitoring of Dynamic Events. Anal. Chem. 1988, 60, 652–656. 10.1021/ac00158a010.

[ref27] Garrido J. M. P. J.; Delerue-Matos C.; Borges F.; Macedo T. R. A.; Oliveira-Brett A. M. Voltammetric oxidation of drugs of abuse: I. Morphine and metabolites. Electroanalysis 2004, 16, 1419–1426. 10.1002/elan.200302966.

[ref28] Goucke C. R.; Hackett L. P.; Ilett K. F. Concentrations of morphine, morphine-6-glucuronide and morphine-3-glucuronide in serum and cerebrospinal fluid following morphine administration to patients with morphine-resistant pain. Pain. 1994, 56, 145–149. 10.1016/0304-3959(94)90088-4. 8008404

[ref29] Heiskanen T.; Langel K.; Gunnar T.; Lillsunde P.; Kalso E. A. Opioid Concentrations in Oral Fluid and Plasma in Cancer Patients with Pain. J. Pain Symptom. Manage. 2015, 50, 524–532. 10.1016/j.jpainsymman.2014.09.004. 25242020

[ref30] Drummer O. H. Postmortem toxicology of drugs of abuse. Forensic Sci Int. 2004, 142, 101–113. 10.1016/j.forsciint.2004.02.013. 15172074

[ref31] Jahanbakhshi M. In situ synthesis of rhodium nanoparticles - Mesoporous carbon hybrid via a novel and facile nanocasting method for simultaneous determination of morphine and buprenorphine. Mater. Sci Eng C. 2019, 97, 479–485. 10.1016/j.msec.2018.12.019. 30678935

[ref32] Gómez L.; Escobar M.; Peñuela O. Standardization of a protocol for obtaining platelet rich plasma from blood donors; a tool for tissue regeneration procedures. Clin. Lab. 2015, 61, 973–980. 10.7754/Clin.Lab.2015.141141. 26427141

[ref33] Tuck M. K.; Chan D. W.; Chia D.; Godwin A. K.; Grizzle W. E.; Krueger K. E.; Rom W.; Sanda M.; Sorbara L.; Stass S.; Wang W.; Brenner D. E. Standard Operating Procedures for Serum and Plasma Collection: Early Detection Research Network Consensus Statement Standard Operating Procedure Integration Working Group. J. Proteome Res. 2016, 14, 51–55. 10.1089/bio.2015.0059. PMC2655764 19072545

[ref34] Basiri F.; Taei M. Application of spinel-structured MgFe2O4 nanoparticles for simultaneous electrochemical determination diclofenac and morphine. Microchim. Acta 2017, 184, 155–162. 10.1007/s00604-016-1995-0.

[ref35] Dehdashtian S.; Gholivand M. B.; Shamsipur M.; Kariminia S. Construction of a sensitive and selective sensor for morphine using chitosan coated Fe3O4 magnetic nanoparticle as a modifier. Mater. Sci. Eng. C. 2016, 58, 53–59. 10.1016/j.msec.2015.07.049. 26478286

[ref36] Leow K. P.; Wright A. W. E.; Cramond T.; Smith M. T. Determination of the serum protein binding of oxycodone and morphine using ultrafiltration. Ther. Drug Monit. 1993, 15, 440–447. 10.1097/00007691-199310000-00014. 8249052

[ref37] Höllt V.; Teschemacher H. Hydrophobic interactions responsible for unspecific binding of morphine-like drugs. Naunyn-Schmiedeberg’s Arch. Pharmacol. 1975, 288, 163–177. 10.1007/BF00500524. 240130 PMC13100346

[ref38] Judis J. Binding of codeine, morphine, and methadone to human serum proteins. J. Pharm. Sci. 1977, 66, 802–806. 10.1002/jps.2600660615. 874779

[ref39] Vree T. B.; Verwey-Van Wissen C. P. W. G. M. Pharmacokinetics and metabolism of codeine in humans. Biopharm. Drug Dispos. 1992, 13, 445–460. 10.1002/bdd.2510130607. 1391681

[ref40] Pöyhiä R.; Seppälä T. Liposolubility and Protein Binding of Oxycodone in Vitro. Pharmacol. Toxicol. 1994, 74, 23–27. 10.1111/j.1600-0773.1994.tb01068.x. 8159633

[ref41] Trouillon R.; Combs Z.; Patel B. A.; O’Hare D. Comparative study of the effect of various electrode membranes on biofouling and electrochemical measurements. Electrochem. Commun. 2009, 11, 1409–1413. 10.1016/j.elecom.2009.05.018.

[ref42] Peltonen A.; Etula J.; Seitsonen J.; Engelhardt P.; Laurila T. Three Dimensional Fine Structure of Nanometer Scale Nafion Thin Films. ACS Appl. Polym. Mater. 2021, 3, 1078–1086. 10.1021/acsapm.0c01318.

[ref43] Gekle M. Renal tubule albumin transport. Annu. Rev. Physiol. 2005, 67, 573–594. 10.1146/annurev.physiol.67.031103.154845. 15709971

[ref44] Moisala A.; Nasibulin A. G.; Brown D. P.; Jiang H.; Khriachtchev L.; Kauppinen E. I. Single-walled carbon nanotube synthesis using ferrocene and iron pentacarbonyl in a laminar flow reactor. Chem. Eng. Sci. 2006, 61, 4393–4402. 10.1016/j.ces.2006.02.020.

[ref45] Kaskela A.; Nasibulin A. G.; Timmermans M. Y.; Aitchison B.; Papadimitratos A.; Tian Y.; Zhu Z.; Jiang H.; Brown D. P.; Zakhidov A.; Kauppinen E. I. Aerosol-synthesized SWCNT networks with tunable conductivity and transparency by a dry transfer technique. Nano Lett. 2010, 10, 4349–4355. 10.1021/nl101680s. 20863125

